# Self-care practices among hypertensive patients at the primary health care centers in Unaizah city, Saudi Arabia

**DOI:** 10.3389/fmed.2024.1290670

**Published:** 2024-12-04

**Authors:** Bahia Galal Abd El-Razik Siam, Hind Kaliefah Aldakeel, Awatif M. Alrasheeday, Salman H. Alsaqri, Bushra Alshammari, Farhan Alshammari

**Affiliations:** ^1^Medical-Surgical Nursing Department, College of Nursing, Hail University, Hail, Saudi Arabia; ^2^Primary Health Nursing Department, College of Nursing, Hail University, Hail, Saudi Arabia; ^3^King Saud Hospital, Onizh, Al Qassiem, Saudi Arabia; ^4^Nursing Administration Department, College of Nursing, Hail University, Hail, Saudi Arabia; ^5^Pharmaceutics Department, College of Pharmacy, Hail University, Hail, Saudi Arabia

**Keywords:** hypertensive, patients, primary health care centers, Saudi Arabia, self-care practices

## Abstract

**Background:**

This study assessed the self-care practices among hypertensive patients at the primary health centers in Unaizah city, Saudi Arabia.

**Methods:**

This cross-sectional study was conducted in 2023, at all primary healthcare centers in Unaizah city. A total of 372 hypertensive patients, aged 18 and older, who had been on follow-up care for at least 6 months were randomly selected using a cluster sampling method. Data regarding socio-demographics, health history, and hypertensive self-care practices (H-SCALE) were collected using a validated questionnaire.

**Results:**

The mean age of the participants was 41.4 ± 9.6 years. Of them, 34.1% were males and 65.9% were females. Only 14.0% were smokers, 55.9% had hypertension for less than 1 year, and 53.8% did not monitor their blood pressure. None of them was addicted to any substance or using alcohol. The most commonly associated chronic disease among the participants was hyperlipidemia (61.3%), followed by diabetes mellitus. The participants were most likely reported engaging in weight management-related self-care activities, followed by healthy diet activities, and medication adherence. The H-SCALE scores increased as the frequency of engagement in self-care activities increased. There were significant differences in H-SCALE scores based on age, gender, education level, and employment status.

**Conclusion:**

Engaging in self-care activities is associated with better self-care behavior. Age, gender, education level, and employment status are significant predictors of hypertension self-care behaviors. Healthcare providers should educate hypertensive patients on blood pressure monitoring and integrated management of hypertension and other chronic diseases.

## Introduction

Hypertension (HTN) is a major cause of premature death worldwide (1). In addition, more than one billion people worldwide suffer from HTN, with two-thirds of these individuals living in low-income nations. Furthermore, approximately one in five adults with HTN has it under control globally (2). Alarmingly, it is predicted that 1.56 billion adults will have HTN by 2025 (3). Uncontrolled HTN can result in kidney failure, blindness, blood vessel rupture, cognitive impairment, heart attack, and ultimately heart failure and stroke (4). Similar to other nations, the Kingdom of Saudi Arabia (KSA) is experiencing an increase in HTN, which must be treated, because it has substantial health implications for the population (5). A countrywide survey of HTN in Saudi Arabia revealed that 15.2% of the population is hypertensive and an additional 40.6% is borderline hypertensive (6).

In fact, the population HTN risk increases with advancing age, diabetes, obesity, sedentary lifestyle, and hypercholesterolemia (7). In Saudi Arabia, numerous factors, including the shift in Saudi Arabia’s lifestyle toward urbanization, unhealthful eating patterns, and obesity are blamed for this rise in the prevalence of HTN (8). Although the precise cause of HTN is unknown, research shows that the risk of HTN is increased by several modifiable factors, including unhealthy alcohol use, physical inactivity, high salt intake, tobacco use, and a high body mass index. Avoiding these risk factors are a key component of self-care practice, which is viewed as a significant action carried out by an individual to improve health or prevent illness (9).

Self-care practices have proven to be an effective and affordable method for preventing and managing HTN and its symptoms. Self-care for HTN include taking medications as prescribed, eating a healthy diet, exercising every day, limiting alcohol intake, quitting smoking, losing weight, self-monitoring blood pressure, getting frequent checkups, and minimizing stress without the use of drugs (10, 11). In Saudi Arabia, Only a few studies have partially explored Self-care practices of hypertensive patients, which was limited to the construct of self-care management. Neminqani et al. (12) determined the level of self-management practices and its associated factors among 124 hypertensive patients in the KSA. The findings revealed that being young, middle class, university educated, and being a woman was found to be associated with a higher level of self-care management. In addition, exercise to reduce weight was found to be one of the lowest performed activities to manage hypertension. In that study, 45.2% of the participants had stopped smoking after being diagnosed with hypertension. Furthermore, the researcher found that being diagnosed with hypertension for more than 5 years was associated with a significantly higher level of self-care management.

A recent cross-sectional analytical study on 419 hypertensive patients conducted in a tertiary care cardiac center in the Kingdom of Saudi Arabia (KSA) revealed that 43% of participants reported they checked their blood pressure at least once a week. All individuals claimed abstinence from alcohol, and 86.8% followed their medication regimen. Physical exercise, weight control, and a healthy diet all had low prevalence rates of 23, 19, and 18%, respectively (13). However, to prevent and decrease the consequences of HTN, the self-care practices should be improved. Therefore, the current study was conducted to assess the self-care practices among hypertensive patients at the primary health centers in Unaizah city, Saudi Arabia.

## Materials and methods

### Study design, period and setting

This cross-sectional study was carried out in 2023, at all public primary healthcare centers in Unaizah city of Qassim region, Saudi Arabia, which offer the care and follow-up services for hypertensive patients. In Unaizah city, there are 16 primary healthcare centers, which offer medical services for patients with chronic diseases, including patients with HTN, who need lifelong care, follow-up and medication supply.

### Study participant, sample size calculation and sampling technique

A convenient sample consisted of all available patients diagnosed with hypertension and visited primary healthcare centers, over a one-month duration were included. The estimated number of documented cases diagnosed with hypertension in Unaizah city and served by the primary healthcare centers was about 7,984 patients at the time of study. Based on the estimated target population and to achieve a 95% confidence level, a minimum of 367 hypertension patients were needed to carry out the study based on the sample size estimation using the Charan and Biswas (14) formula. However, the responses were gained from 372 hypertension patients from the study setting.

### Procedure

Data collection was carried out through the distribution of the questionnaire to the participants (the interview conducted during the scheduled follow-up visit in the PHC centers). Each patient took from 20 to 30 min to measure vital signs, weight, and filling the questionnaire. This was done by the researchers and under supervision of the specialist who is following the case in the health care center.

### Data collection tools

In the current study, all data were gathered using an interview-based questionnaire that consist of three parts as follows: (1) socio-demographic characteristics of the study participants; (2) health history associated with HTN of the study participants; and (3) the hypertensive self-care activity level effect (H-SCALE) of the study participants.

### Assessment of self-care practices among hypertensive patients

In the current study, the hypertensive self-care activity level effect scale (H-SCALE) was used to assess the self-care practices among the hypertensive patients. The H-SCALE was developed and adapted in previous studies (15, 16). The H-SCALE consists of 28 items measuring five self-care activities (subscales); three items measuring medication adherence, 12 items assess practices related to eating a healthy diet, physical activity was assessed with two items, two for smoking, and 10 items assessing weight management. The three items for alcohol intake were excluded respecting the country culture. The questionnaire was in English language, then translated and administered in Arabic language. The questionnaire language appropriateness, content validity, question comprehensibility, and refining were achieved by six experts from relevant fields before actual distribution among the participants. The reliability, consistency, and stability of the questionnaire was tested using the Cronbach alpha coefficient (*α* = 0.85).

### Scoring

Each item is rated from zero to seven reflecting how frequently this self-care activity was implemented by the patient during the last week, zero indicates poor adherence to self-care practices, while higher scores indicating good self-care, except for smoking as smoking is bad behavior (0 means the patient is completely non-smoker). Each item was rated from (0 to 7). The total H-SCALE score is the sum of all items; score in each subscale.

### Pilot study

Pilot study was carried out on 20 participants; the questionnaire and data collection process were modified according to the result of the pilot study.

### Statistical analysis

Following data collection, the Statistical Package for Social Sciences (SPSS) program 26 was used to analyze the data representing the results in accordance with the type of information in tables and graphs. Descriptive statistics were used to illustrate the socio-demographic characteristics, HTN health history of the participants, and the self-care practices performed by hypertensive patients. Moreover, non-parametric tests were used to identify any significant difference in the total self-care practices scale based on participants’ socio-demographic variables, as data are not following the normality.

### Results

A total of 372 of participants were included in the final analysis; of them, 34.1% were males and 65.9% were females. The mean age of the study participants was 41.4 ± 9.6 years. In addition, 51.9% of the participants were aged between 40 to less than 60 years, and large percentage 72% of them were married. The most common level of education was a bachelor’s degree (26.9%), 61.3% of the participants were employed, and 46.2% reported having enough to some extent monthly income as shown in Table 1.

**Table 1:** Socio-demographic characteristics of the study participants.

Socio-demographic variables	*N* = 372	100%
Age (years)		
<25 years	40	10.7%
25 to less than 40 years	125	33.6%
40 to less than 60 years	193	51.9%
60 years or more	14	3.8%
Mean ± SD: 41.4 ± 9.6		
Gender		
Males	127	34.1%
Females	245	65.9%
Marital status		
Single	56	15.1%
Married	268	72.0%
Divorced	32	8.6%
Widowed	16	4.3%
Level of education		
Illiterate	4.0	1.1%
Primary/can read	16	4.3%
Intermediate education	28	7.5%
Secondary education	92	24.7%
Vocational education	52	14.0%
Bachelor’s degree	100	26.9%
Master’s degree and above	80	21.5%
Employment status		
Employee	228	61.3%
Housewife	44	11.8%
Retired	44	11.8%
Student	20	5.4%
Unemployed	36	9.7%
Monthly income		
Enough income	112	30.1%
Enough to some extent	172	46.2%
Not enough	88	23.7%

As shown in Table 2, the duration of HTN varied among the study participants, with 55.9% of them having HTN for less than 1 year. Only 14.0% of them were currently smokers. In addition, more than half of the study participants 53.8% did not monitor their blood pressure, while 29.0% were monitoring their blood pressure one to two times per week. None of the study participants was addicted to any drug or substance, and none of them used alcohol. The most associated chronic diseases among the study participants were hyperlipidemia (61.3%), diabetes mellitus (16.1%), followed by bronchial asthma (7.5%), and renal diseases (6.5%). Furthermore, the mean systolic and diastolic blood pressure (mmHg) of the study participants were 140.3 ± 5.2, and 84.9 ± 3.8, respectively.

**Table 2:** Health history associated with hypertension of the study participants.

Health history associated with hypertension	*N* = 372	100%
Duration of hypertension		
Less than 1 year	208	55.9%
1 year to 5 years	56	15.1%
5 to 10 years	32	8.6%
More than 10 years	76	20.4%
Current smoking status		
Currently smoker	52	14.0%
Not smoker	320	86.0%
Frequency of blood pressure monitoring per week		
1 to 2 times	108	29.0%
3 to 4 times	32	8.6%
5 to 6 times	20	5.4%
7 or more times	12	3.2%
I do not monitor	200	53.8%
Are you addicted to any drug or substance?		
No	372	100%
History of alcohol intake		
No	372	100%
Associated chronic diseases		
Bronchial asthma	28	7.5%
Diabetes mellitus	60	16.1%
Renal diseases	24	6.5%
Hyperlipidemia	228	61.3%
Others	28	7.5%
Blood pressure (mmHg)		
Systolic blood pressure	Mean ± SD	140.3 ± 5.2
Diastolic blood pressure	Mean ± SD	84.9 ± 3.8

**Figure 1:**
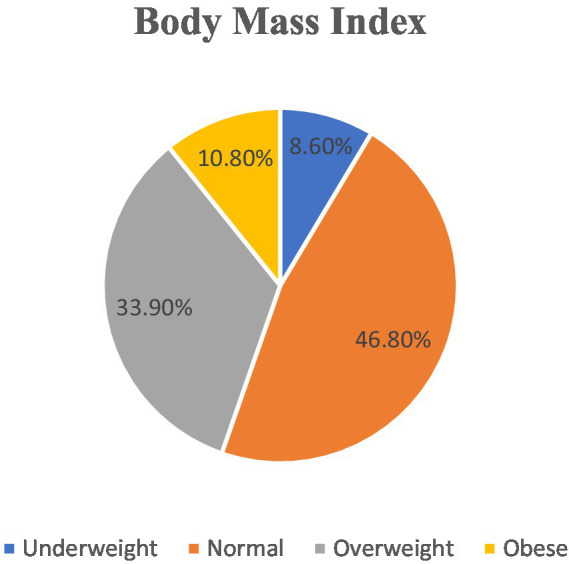
Body mass index of the participants.

Figure 1 shows that 46.8% of the participants had normal body mass index, 33.9 of them were overweight, while 8.6 of them were underweight.

Table 3 shows that 63.0% of the participants are considered nonsmokers as they did not smoke any cigarettes for the last 7 days. 45.0% of the participants reported nonadherence to medication and physical activity. The overall mean score of the H-SCALE among the study participants was (47.7 ± 23.5). The hypertensive patients were most likely reported engaging in weight management-related self-care activities (the highest mean scores: 21.8 ± 10.3), followed by healthy diet-related self-care activities (the mean scores were 16.7 ± 11.5), and medication adherence (the mean scores were 5.2 ± 6.6). Smoking and physical activity-related self-care activities were reported less frequently (the mean scores for both were 2.5 ± 2.5, and 2.3 ± 2.6, respectively).

**Table 3:** Frequencies and mean scores of the hypertensive self-care activity level effect (H-SCALE) of the study participants during the past 7 days.

H-SCALE variables	0 time		1 time		2 times		3 times		4 times		5 times		6 times		7 times		Scale
	N	%	N	%	N	%	N	%	N	%	N	%	N	%	N	%	Mean ± SD
Medication adherence	504	45%	208	19%	104	9.3%	80	7.1%	56	5%	24	2.1%	16	1.4%	124	11.1%	5.2 ± 6.6
Healthy diet	1,208	29%	1,332	33%	736	18%	360	9%	208	5%	84	2%	60	1%	104	3%	16.7 ± 11.5
Physical activity	336	45%	188	25%	100	13%	68	9%	16	2%	20	3%	4.0	1%	12	2%	2.3 ± 2.6
Smoking	472	63%	112	15%	100	13%	28	4%	4.0	1%	8.0	1%	0.0	0%	20	3%	2.5 ± 2.5
Weight management	0.0	0.0%	1,592	43%	812	22%	648	17%	388	10%	280	8%	0.0	0%	0.0	0%	21.8 ± 10.3
Total	2,520	24%	3,432	33%	1,852	18%	1,184	11%	672	6%	416	4%	80	1%	260	3%	47.7 ± 23.5

Table 4 revealed significant differences in the total H-SCALE scores based on age categories, ender, level of education, and employment status of the study participants. While there was no significant relation between the total H-SCALE scores based on marital status and monthly income. Males, participants aged 25 to less than 40 years, and those who had a bachelor’s degree had much higher average ranks than others did, while employed participants had a significantly lower mean rank compared to retired participants.

**Table 4:** Differences in the H-SCALE based on the participants socio-demographic characteristics.

Socio-demographic variables		H-SCALE		*p*-value
		*N*	Mean rank	
Age (years)	<25 years	40	166.10	<0.001^*^
	25 to less than 40 years	125	197.20	
	40 to less than 60 years	193	169.11	
	60 years or more	14	194.40	
Gender	Males	127	208.25	0.005^*^
	Females	245	175.09	
Marital status	Single	56	156.07	0.057
	Married	268	193.25	
	Divorced	32	169.00	
	Widowed	16	215.00	
Level of education	Bachelor’s degree	100	212.66	0.005^*^
	Intermediate education	28	195.93	
	Master’s degree and above	80	188.20	
	Illiterate	4	50.50	
	Primary education	16	193.50	
	Secondary education	92	178.15	
	Vocational education	52	151.58	
Employment status	Employed	228	181.76	<0.001^*^
	Housewife	44	122.50	
	Retired	44	259.59	
	Student	20	199.70	
	Unemployed	36	198.06	
Monthly income	Enough income	112	189.36	0.364
	Enough to some extent	172	178.69	
	Not enough	88	198.14	

^*^Significant (*p* < 0.05).

## Discussion

The findings of the current study provide information about the health care practices among patients with HTN Unaizah city, Saudi Arabia.

The findings showed that women represented more than two thirds of the participants. Over half of the participants were in the 40–60-year age range with a mean age of 41.4 ± 9.6 years, and a significant proportion were married. Nearly two thirds of the participants were working, nearly half reported having enough money each month, and the most prevalent level of education was a bachelor’s degree (26.9%).

The main findings of the current study revealed that more than one third of the participants were overweight, the participants were most likely reported engaging in weight management-related self-care activities, followed by healthy diet-related self-care activities, and medication adherence. In addition, the H-SCALE scores increased as the frequency of engagement in self-care activities increased. As regards the current health history associated with HTN, the results reveal that the duration of HTN varied among the participants, more than half of the participants reported having HTN for less than 1 year. This suggests that HTN may have been recently diagnosed in many of the participants, and they may not have had sufficient time to manage their condition effectively. Abdisa et al. (17) found that almost half of the patients were diagnosed with HTN for less than 5 years.

Concerning smoking, most participants were not smokers. As a result of the low percentage of smokers as reported in the sample selected, this may have the greatest impact on the decrease in the extent of interest in reducing smoking, as it is not on the list of priorities and interests of the selected sample of hypertensive patients. This finding is inconsistent with an earlier study, which reported that smoking and substance abuse are risk factors for HTN and other chronic diseases (18). Smoking cessation is also essential, as smoking is a significant risk factor for HTN and other chronic diseases (19).

More than half of the participants reported not monitoring their blood pressure at all. This result goes in the same line with Abdisa et al. (17) who stated that more than half of participants had a history of missed follow-ups. However regular monitoring of blood pressure is essential in managing HTN and preventing its complications, it will be effective only when the patients provide regular feedback to the healthcare providers in order to provide special care. These findings are supported by another previous studies Tucker et al. (20) and Rhee et al. (21) who demonstrated that self-monitoring of blood pressure alone without provision of data to the healthcare team and advising back to the patient has limited impact on hypertension control.

Near two thirds of the participants reported having hyperlipidemia which is a major risk factor of hypertension and cardiovascular diseases. Ongoing therapy is necessary for hypertension, as it is a chronic illness. To ensure optimal control of HTN and reduce the likelihood of consequences, regular blood pressure monitoring is crucial (22). The other associated chronic disease was diabetes mellitus, followed by asthma and renal diseases. This finding is supported by previous studies which demonstrated that HTN is often associated with other chronic diseases, particularly diabetes mellitus and renal diseases (23, 24). The management of these chronic diseases should be integrated with HTN management to improve overall health outcomes.

Self-care practices are crucial for managing HTN and can lower the likelihood of complications from the illness. Patients were most likely reported engaging in weight management-related self-care activities, followed by healthy diet-related self-care activities, and medication adherence. This finding goes in the same line with Konlan and Shin (25) who reported that diet modification and medication adherence are the most common self-care activities among hypertensive patients. Farag et al. (26) also, stated that weight management is an important aspect of HTN management as obesity is a significant risk factor for the development of HTN and its complications. Physical activity and smoking-related self-care activities were reported less frequently. This finding is concerning, as physical activity and smoking cessation are crucial in the management of HTN and reducing the risk of cardiovascular diseases.

Regular exercising is one of the activities that require commitment, and patients may suffer from difficulty in adhering to it, especially with the fluctuating weather and intense heat even in the evening hours for several months of the year in Saudi Arabia. Also, the culture of exercising is not sufficiently widespread in societies, especially in Arab countries, which may reflect the reason for the low extent of blood pressure patients’ commitment to regular exercise.

As the frequency of engagement in self-care activities increased, the total H-SCALE scores also increased, indicating better self-care behavior. In the current study, the mean H-SCALE score of 47.73 indicated that on average, participants in this study engaged in a moderate level of HTN self-care activities. This finding is consistent with previous research that has shown that hypertensive patients who engage in regular self-care activities have better blood pressure control and lower risk of complications (5, 27). The higher scores of H-SCALE indicates better self-care behavior (28). A recent study has reported a wide range of engagement levels in self-care activities among individuals with HTN (27). This variability can be associated with factors such as age, gender, education level, and access to healthcare services. The findings of this study align with the existing literature, suggesting that engagement in HTN self-care activities is heterogeneous among individuals. Conversely, another study has reported a narrower range of H-SCALE scores and less variability among participants (29). That study had a larger sample size, and may have controlled certain factors, such as socioeconomic status or healthcare access leading to a more homogeneous sample. The differences in findings could also be due to variations in study design, sample size, and measurement tools used to assess self-care activities.

The current study findings indicated that male participants had higher H-SCALE scores than female participants. Possible explanations for the higher H-SCALE scores among male participants in the include differences in healthcare-seeking behaviors. Men are less likely than women to pursue healthcare services including preventive care and chronic disease management. Results of past research on gender differences in self-care behaviors for HTN have been contradictory. It is also possible that social and cultural factors contributed to the observed gender differences. Some studies have found that women engage in more self-care activities than men and they had more awareness about HTN (30, 31), while other has reported than women with HTN were less to engage in self-care activities than men (32).

The current research findings suggest that sociodemographic factors such as age, gender, education level, and employment status may have significant effects on HTN self-care activity level, while employed participants had a significantly lower mean rank compared to retired participants. These findings are consistent with some previous studies that have also found a positive association between age and HTN self-care behaviors. For example, AlGhurair et al. (33) found that older age was associated with better self-monitoring of blood pressure and medication adherence. The finding that higher education levels were associated with better HTN self-care behaviors is also consistent with previous research. For instance, a study by Kim et al. (34) found that higher education levels were associated with better medication adherence among hypertensive patients. Similarly, a study by Chiang et al. (35) who reported that higher education levels were associated with better self-care behaviors such as exercise and diet in hypertensive patients. The finding that employed participants had lower H-SCALE scores than retired, student, and unemployed participants is somewhat surprising and appears to contradict previous research. Many studies have reported that employment status is not a significant predictor of HTN self-care behaviors (34, 35). However, it is possible that the current study’s finding may be due to the specific characteristics of the employed participants in this study. For example, the employed participants may have had more demanding work schedules or job-related stress, which may have negatively impacted their ability to engage in HTN self-care activities.

Further future studies are recommended to investigate the factors that contribute to the variability in H-SCALE scores, and to examine the effectiveness of personalized interventions in improving HTN self-care activities. Moreover, a health promotion program is recommended for same participants in order to improve their self-care practices which could impact their quality of lives and reduce possible complications.

It should be noted that the current study’s findings are based on self-reported data, which may be subject to recall bias and social desirability bias. Additionally, the study used a cross-sectional design, which limits the ability to draw causal inferences. In conclusion, the current study’s findings suggest that age and education level are significant predictors of hypertension self-care activities, while the relationship between employment status and hypertension self-care behaviors requires further investigation. However, there was no significant difference in hypertension self-care activities H-SCALE scores based on patients’ marital status or monthly income.

### Strength and limitations

A limitation of this study pertains to its cross-sectional design, affecting the generalizability of outcomes. In addition, the current study’s findings are based on self-reported data, which may be subject to recall bias and social desirability bias. Conversely, a strength of this study lies in being the first to show the self-care practices among hypertensive patients at the primary health care centers in Unaizah City, Saudi Arabia, and its representative sample size.

## Conclusion

Hypertensive patients are more likely to engage in diet-related self-care activities, medication usage, and weight management, while physical activity and smoking-related self-care activities are reported less frequently. Engaging in self-care activities is associated with better self-care behavior, as indicated by a higher H-SCALE score. Weight management is an essential aspect of self-care behavior among hypertensive patients, whereas smoking cessation may require additional support and resources. Healthcare providers should educate hypertensive patients on the importance of engaging in self-care activities and provide them with the necessary resources and support to improve their self-care behavior. The wide range of H-SCALE scores and the considerable variability among individuals in this study highlight the need for personalized interventions to promote hypertension self-care activities. Tailored programs that consider individual differences and target specific areas of improvement in self-care engagement can potentially lead to better hypertension management and overall health outcomes.

## Data Availability

The raw data supporting the conclusions of this article will be made available by the authors, without undue reservation.
